# Limited association between disinfectant use and either antibiotic or disinfectant susceptibility of *Escherichia coli* in both poultry and pig husbandry

**DOI:** 10.1186/s12917-019-2044-0

**Published:** 2019-09-02

**Authors:** Helder Maertens, Koen De Reu, Evelyne Meyer, Els Van Coillie, Jeroen Dewulf

**Affiliations:** 1 Flanders Research Institute for Agriculture, Fisheries and Food (ILVO), Technology and Food Science Unit, Brusselsesteenweg 370, 9090 Melle, Belgium; 2 Veterinary Biochemistry Unit, Department of Pharmacology, Toxicology and Biochemistry, Faculty of Veterinary Medicine, Ghent University, Salisburylaan 133, 9820 Merelbeke, Belgium; 3 Veterinary Epidemiology Unit, Department of Reproduction, Obstetrics and Herd Health, Faculty of Veterinary Medicine, Ghent University, Salisburylaan 133, 9820 Merelbeke, Belgium

**Keywords:** Antibiotic resistance, Disinfectant susceptibility, *E. coli*, Broiler flocks, Pig herds, Biocidal products, Disinfection

## Abstract

**Background:**

Farm disinfectants are widely used in primary production, but questions have been raised if their use can select for antimicrobial resistance. The present study examined the use of disinfectants in poultry and pig husbandry and its contribution to the antibiotic and disinfectant susceptibility of *Escherichia coli* (*E. coli*) strains obtained after cleaning and disinfection. On those field isolates antibiotic susceptibility was monitored and susceptibility to commonly used active components of farm disinfectants (i.e. glutaraldehyde, benzalkoniumchloride, formaldehyde, and a formulation of peracetic acid and hydrogen peroxide) was tested.

**Results:**

This study showed a high resistance prevalence (> 50%) for ampicillin, sulfamethoxazole, trimethoprim and tetracycline for both production animal categories, while for ciprofloxacin only a high resistance prevalence was found in broiler houses. Disinfectant susceptibility results were homogenously distributed within a very small concentration range. Furthermore, all *E. coli* strains were susceptible to in-use concentrations of formaldehyde, benzalkoniumchloride and a formulation of peracetic acid and hydrogen peroxide, indicating that the practical use of disinfectants did not select for disinfectant resistance. Moreover, the results showed no indications for the selection of antibiotic resistant bacteria through the use of disinfectants in agricultural environments.

**Conclusion:**

Our study suggests that the proper use of disinfectants in agricultural environments does not promote antibiotic resistance nor reduce *E. coli* disinfectant susceptibility.

**Electronic supplementary material:**

The online version of this article (10.1186/s12917-019-2044-0) contains supplementary material, which is available to authorized users.

## Background

Biocidal products are frequently used chemicals with the aim to inactivate microorganisms [1] harmful to human or animal health. Biocides used for veterinary hygiene purposes are applied to disinfect materials and surfaces associated with the housing or transportation of animals. They play a crucial role in preventing and controlling the transmission of infections within and between herds, which is an important aspect of on-farm biosecurity.

Despite the increasing use of disinfectants, bacteria seem to remain susceptible to these disinfection products when used correctly. Their in-use concentrations are normally far above the minimum inhibitory concentration (MIC) of wildtype isolates [2], as opposed to antibiotics for which MICs are generally closer to concentrations used in practice. Furthermore, as disinfectants generally contain more than one type of active component each with a different antimicrobial mode of action [1] and as they have no specific microbial target, the development of resistance at the level of in-use concentrations is thought to be highly unlikely [3, 4].

However, in practice, disinfectants can be found at lower concentrations due to underdosing, or due to residual organic debris as a result of insufficient cleaning, or due to dilution by remaining rinsing water. Under such conditions, bacteria are exposed to subinhibitory disinfectant concentrations, which could lead to a selection of strains with a reduced susceptibility to disinfectants [5]. Moreover, concerns have been raised about a possible selection of antibiotic resistant bacteria through the use of disinfectants. The emergence of reduced susceptibility of bacteria to antimicrobials (disinfectants and antibiotics) induced by disinfectants has been demonstrated in vitro*.* Laboratory-based adaptation experiments have shown that step-wise exposure of initially susceptible bacteria to subinhibitory concentrations of benzalkoniumchloride, chlorhexidine, triclosan and some commercial disinfectants may lead to decreased susceptibility to either antibiotics or disinfectants [6–9]. Recent studies investigated the disinfectant susceptibility of bacteria isolated from live-stock and its environment [10–14] or evaluated the correlation [2, 15] or association [16] between antibiotic resistance and a decreased susceptibility to disinfectants. However, in marked contrast to the in vitro reports, no evidence that the use of disinfectants selects for antimicrobial resistance under practical conditions was found. Furthermore, there are only few studies on the susceptibility of bacteria isolated from livestock environments after cleaning and disinfection and most studies on disinfectant susceptibility examined minimum inhibitory concentrations (MICs) but did not evaluate the lethal effects of the disinfectants by determining the minimum bactericidal concentration (MBC).

Therefore, the current study aimed at filling these gaps by examining the use of disinfectants in poultry and pig husbandry and its contribution to the antibiotic and disinfectant susceptibility of *Escherichia coli* (*E. coli*) isolates.

## Results

### Biosecurity

The scores of the different categories of the biocheck scoring system are listed in Table 1. The average external and internal biosecurity scores for broiler farms were 66.9 (range 54.0–78.0) and 61.0 (range 40.0–80.0), respectively and for pig farms 69.0 (range 57.0–87.0) and 65.9 (range 46.0–88.0), respectively.

**Table 1:** Descriptive results of the different aspects of external and internal biosecurity scores for 25 broiler farms and 21 pig farms

Broiler farms	Min.	Mean	Max.	Pig farms	Min	Mean	Max
*External biosecurity*	*54*	*67*	*78*	*External biosecurity*	*57*	*69*	*87*
Purchase of one day old chicks	37	69	100	Purchase of animals and semen	78	94	100
Exports of live animals	51	67	92	Transport of animals, removal of manure and dead animals	52	71	100
Feed and water supply	43	57	96	Feed, water and equipment supply	30	44	67
Removal of manure and dead animals	26	74	90	Personnel and visitors	47	68	88
Entrance of visitors and personnel	41	70	90	Vermin and bird control	50	72	100
Supply of materials	0	43	56	Environment and region	10	41	100
Infrastructure and biological vectors	65	83	100				
Location of the farm	15	60	81				
*Internal biosecurity*	*40*	*61*	*80*	*Internal biosecurity*	*46*	*66*	*88*
Disease management	56	78	88	Disease management	40	72	100
Cleaning and disinfection	28	52	71	Farrowing and suckling period	14	62	100
Materials and measures between compartments	0	55	100	Nursery unit	50	80	100
				Fattening unit	36	84	100
				Measures between compartments and the use of equipment	18	57	100
				Cleaning and disinfection	18	54	88
*Overall biosecurity*	*50*	*65*	*74*	*Overall biosecurity*	*54*	40	72

### Cleaning and disinfection practices

Descriptive results of the different cleaning and disinfection protocols carried out at the 25 broiler farms and the 21 pig nursery units are listed in Table 2. Results showed that the most complete cleaning protocol, consisting of dry cleaning followed by soaking (with water), cleaning with a cleaning product and rinsing of the cleaning product is more applied at the broiler houses compared to the pig nursery units. The greatest variation in disinfection protocols was seen in broiler houses. For the pig nursery units, disinfection was always applied by the farmer with 1 disinfectant by fogging (4.8%) or foaming (95.2%). For both sectors, the most frequently used disinfectants consisted of a combination of QACs and glutaraldehyde. In contrast to the broiler house, pig nursery units were less frequently disinfected.

**Table 2:** Descriptive results of the different cleaning and disinfection protocols carried out at 25 broiler houses and 21 pig nursery units

Category	Step / Parameter	Broiler houses (%)	Pig nursery units (%)
Cleaning	**Dry cleaning**	100	14.3
	**Soaking (only water)**	92.0	76.2
	**Cleaning with cleaning product**	92.0	95.2
	**Rinsing**	100	85.7
Disinfection	**Disinfection responsible**		
	Farmer	76.0	100
	Specialist contractor	24.0	0
	**Disinfectants**		
	1 disinfectant used	80.0	100
	≥ 2 disinfectants used	20.0	0
	**Disinfection steps**		
	1 disinfection step	84.0	100
	2 disinfection steps	16.0	0
	**Composition of the used disinfectant during disinfection**		
	QAC-GA	44.0	81.0
	QAC-F-GA	12.0	4.8
	F	12.0	0
	PA-H_2_O_2_	8.0	9.5
	Other:		
	Potassium peroxymonosulfate	4	4.8
	Other combinations	20	0
	**Disinfection method**		
	Fogging	16.0	4.8
	Spraying*	56.0	0
	Foaming	16.0	95.2
	Combined methods (≥ 2 disinfectants)	12.0	0
	**Disinfection frequency**		
	Every production round	96.0	71.4
	Every ≥2 production rounds	4.0	28.6
	**Rinsing**	0	19.0
Period	**Vacancy (range):**	9.5 days (4–28)	5.0 days (2–7)
	Dry cleaning (range)	0.2 days (0–1)	0 days
	Wet cleaning (range)	1.6 days (0–7)	0.3 days (0–1)
	Disinfection (range)	4.6 days (1–21)	1.0 days (0–3)

* Spraying was done by using an orchard sprinkler; F, formaldehyde; PA, peracetic acid; H_2_O_2_, hydrogen peroxide; QAC, quaternary ammonium compound (QACs are a large group of related compounds e.g. benzalkoniumchloride, didecyldimethylammoniumchloride); GA, glutaraldehyde

### Detection of *Escherichia coli*

Approximately 24 h after disinfection, *E. coli* was found in 20.3% (222 of 1095) and 46.0% (229 of 498) samples from poultry and pig farms, respectively. This resulted in 200 and 206 *E. coli* isolates respectively, as in some cases a pure culture could not be obtained. Especially floor cracks (38%), drain holes (48%) and drinking cups (28%) of the sampled broiler houses were positive for *E. coli*. At the pig nursery units, *E. coli* was detected at the floor (50%), concrete wall (24%), synthetic wall (20%), feeding trough (58%), drinking nipples (57%) and pipes (65%).

### Antibiotic susceptibility

**Fig. 1:**
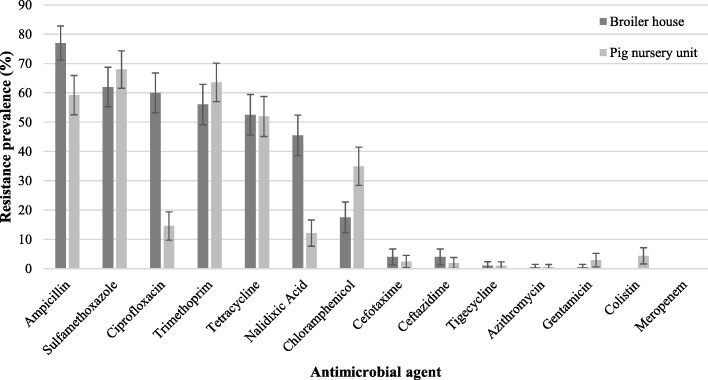
Prevalence of antibiotic resistance (expressed as percentage) of 200 and 206 *E. coli* isolates from 25 broiler houses and 21 pig nursery units respectively. Error bars represent 95% confidence interval

All these *E. coli* isolates were tested for their susceptibility to 14 antibiotics. Their antibiotic resistance prevalence is shown in Fig. 1.

For the *E. coli* isolates from broiler chickens (n = 200), very high levels (> 50%) of antibiotic resistance to ampicillin (77%), sulfamethoxazole (62%), ciprofloxacin (60%), trimethoprim (56%) and tetracycline (53%) were found. A high (> 20–50%) and moderate (> 10–20%) resistance was noted for nalidixic acid (46%) and chloramphenicol (18%), respectively. Resistance towards cefotaxime, ceftazidime, tigecycline, azithromycin and gentamicin was relatively low (≤ 10%). Only 13% of the broiler *E. coli* isolates were susceptible to all tested antibiotics. For *E. coli* isolates (n = 206) from pig nursery units, antibiotic resistance was very high (> 50%) for sulfamethoxazole (68%), trimethoprim (64%), ampicillin (59%) and tetracycline (52%). A high (> 20–50%) antibiotic resistance was found for chloramphenicol with 35%. Resistance to ciprofloxacin and nalidixic acid was moderate with levels of 15 and 12%, respectively. A low level (≤ 10%) of resistance was found for colistin, gentamicin, cefotaxime, cetazidime, tigecycline and azithromycin. Only 21% of the pig herd *E. coli* isolates were susceptible to all antibiotics tested. Multidrug resistance - defined as resistance to three or more classes of antibiotics - was found in 135 (68%) and 130 (63%) *E. coli* field isolates, from broiler houses and pig nursery units, respectively. For both sectors, no *E. coli* isolates were found resistant to all β-lactam antibiotics of the panel (meropenem, cefotaxime, ceftazidime, ampicillin).

### Disinfectant susceptibility

#### Selected isolates

**Fig. 2:**
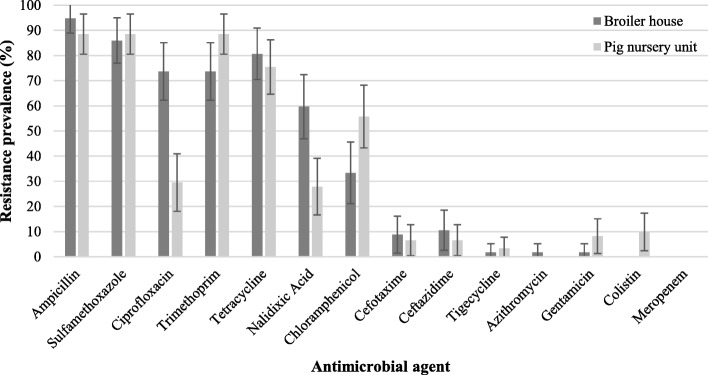
Prevalence of antibiotic resistance (expressed as percentage) of 57 and 61 *E. coli* isolates from 25 broiler houses and 21 pig nursery units respectively, selected for disinfectant susceptibility testing. Error bars represent 95% confidence interval

Antibiotic resistance prevalences of the selected isolates for disinfectant susceptibility testing are available in Fig. 2. Antibiotic resistance profiles of the *E. coli* strains isolated from the same farm differed. Additional file 1 shows this in more detail.

#### MIC and MBC results

**Fig. 3:**
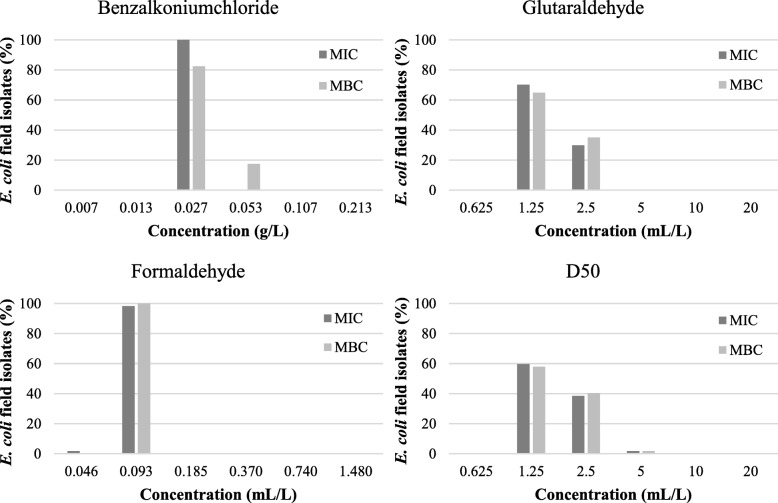
Minimum inhibitory (MIC) and minimum bactericidal concentrations (MBC) of 57 *E. coli* field isolates from broiler houses for 3 active components (benzalkoniumchloride, glutaraldehyde, formaldehyde) and 1 commercial disinfection product (D50: peracetic acid and hydrogen peroxide formulation), expressed as percentages

**Fig. 4:**
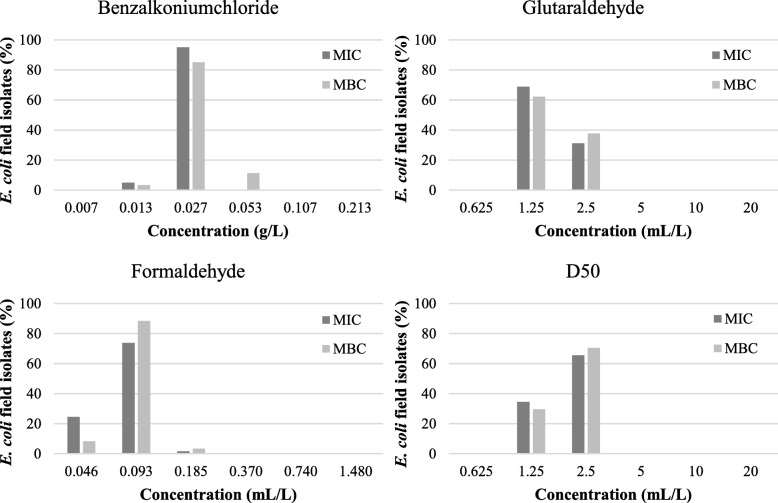
Minimum inhibitory (MIC) and minimum bactericidal concentrations (MBC) of 61 *E. coli* field isolates from pig nursery units for 3 active components (benzalkoniumchloride, glutaraldehyde, formaldehyde) and 1 commercial disinfection product (D50: peracetic acid and hydrogen peroxide formulation), expressed as percentages

Results of the MICs and MBCs of the selected 57 broiler and 61 pig *E. coli* field isolates for the tested disinfectants are given in Fig. 3 and Fig. 4, respectively. For benzalkoniumchloride, MICs of 0.027 g/L and 0.013–0.027 g/L were found for *E. coli* isolates isolated from broiler houses and pig farms, respectively. The MBCs were 0.027–0.053 g/L for isolates from broiler houses and ranged from 0.013 to 0.053 g/mL for isolates from pig nursery units. The MICs and MBCs for glutaraldehyde ranged between 1.25 and 2.5 mL/L for isolates of both sectors. For formaldehyde, a MIC of 0.046–0.093 mL/L was found for isolates from broiler houses while MICs for isolates from pig nursery units ranged from 0.046–0.185 mL/L. The MBC was 0.093 mL/L and between 0.046–0.185 mL/L for isolates from broiler houses and nursery units, respectively. The MICs and MBCs for D50 were between 1.25–5 mL/L and 1.25–2.5 mL/L for isolates of broiler and pig farms, respectively. Most of the MICs and MBCs were the same, demonstrating the bactericidal effect of the active components at the lowest concentration that inhibited growth.

#### Evaluation of MIC and MBC results

After visual examination of the MIC and MBC histograms for both animal species, it was not possible to set a cut-off value separating the *E. coli* field isolates into a disinfectant-susceptible and -resistant population as there was no bi-modal distribution.

### Association between disinfectant use and antibiotic resistance prevalence

In broiler production, significant negative associations were found between the use of peracetic acid and hydrogen peroxide and ampicillin, ciprofloxacin and tetracycline resistance (Table 3). No significant associations were found for the other active components and antibiotics. In pig production, no significant associations between the use of active disinfectant components and antibiotic resistance were found.

**Table 3:** Odds ratios (OR) of significant associations (P-value) between the use of active components of disinfectants and antibiotic resistance in broiler production

Used active components	Antibiotic resistance (prevalence)		
	Ampicillin (0.77)	Ciprofloxacin (0.60)	Tetracycline (0.53)
PA-H_2_O_2_	OR = 0.116 (< 0.001)	OR = 0.290 (0.002)	OR = 0.223 (0.001)

PA, peracetic acid; H_2_O_2_, hydrogen peroxide

### Association between disinfectant use and disinfectant susceptibility

All *E. coli* isolates showed a similar susceptibility to the active components (formaldehyde, benzalkoniumchloride, glutaraldehyde and formulation of peracetic acid and hydrogen peroxide), hence no indications for disinfectant resistance were found and no statistical analysis could be performed.

## Discussion

### Biosecurity and cleaning and disinfection practices

Results of the overall biosecurity at the sampled broiler farms were in line with those of previous Biocheck.UGent questionnaires in Belgium (average biosecurity level of 65 versus 67) [17]. Results for the overall biosecurity at the sampled pig farms, were slightly better (average biosecurity level of 68 versus 61) due to better external and internal biosecurity scores. One of the most important sub-categories of internal biosecurity, i.e. to reduce the risk of pathogen spreading within herds, is the cleaning and disinfection (C&D) score. In the current study, the latter score was comparable to the average Belgian C&D score for both animal categories (52 vs. 56 for broiler farms and 54 vs. 48 for pig farms) indicating that the sampled farms are representative for the average Belgian farm. More importantly, these results indicate that substantial improvements at the level of internal biosecurity and more specifically in cleaning and disinfection, can still be made. The most frequently used active components of disinfectants in both animal species are a combination of QACs and glutaraldehyde, while formaldehyde and a combination of peracetic acid and hydrogen peroxide were also commonly used. This is consistent with a recent study of our group by Maertens et al. (2018) [18] on C&D in Belgian poultry production and supports our choices of active components tested for their susceptibility.

### Antibiotic susceptibility

The *E. coli* field isolates from the sampled broiler farms showed very high resistance for ampicillin, sulfamethoxazole, ciprofloxacin, trimethoprim and tetracycline, which is in line with the report by CODA-CERVA for *E. coli* isolates from Belgian broilers in 2015 [19]*.* The common use of the corresponding antibiotic classes (penicillins, sulfonamides, fluoroquinolones and tetracyclines) in broiler production in Belgium [20], is in line with these high resistance levels. In the CODA-CERVA report [19], very low resistance to ceftazidime and cefotaxime (4.6%) were found, again corroborating our findings. The *E. coli* field isolates from the sampled pig nursery units, showed very high resistance to sulfamethoxazole, trimethoprim, ampicillin and tetracycline. In general, penicillins, the combination of sulphonamides with trimethoprim and tetracyclines are the most commonly used classes of antibiotics in pigs [21] which are strongly correlated to the resistance level [22]. Slightly lower resistance levels to ampicillin, sulfamethoxazole and tetracycline were found *in E. coli* from Belgian pigs in 2015 [19].

### Disinfectant susceptibility

Overall, the MICs and MBCs of the susceptibility tests did not indicate disinfectant resistance as these values showed a homogeneous distribution and no remarkable differences in either parameters were found between the isolates, which is in agreement with Oosterik et al. (2014). Furthermore, our MIC and MBC values of our *E. coli* isolates ranging from 27 to 53 mg/L for benzalkoniumchloride are similar to previous findings. Indeed, Buffet-bataillon et al. (2011) reported MICs for benzalkoniumchloride between 16 and 32 mg/L for 94% of the examined clinical *E. coli* population. In other studies, MICs were found for benzalkoniumchloride of either 32 mg/L for 52.6% of the *E. coli* isolated from retail meats [23] or between 8 and 32 mg/L in avian pathogenic *E. coli* [11]. The MICs and MBCs for formaldehyde ranged between 0.046 to 0.093 mL/L (~ 50 to 101 mg/L). Likewise, Oosterik et al. (2014) reported MICs and MBCs to formaldehyde of 40 to 80 mg/L. The MICs found for glutaraldehyde of 1.25 to 2.5 mL/L (~ 1383 to 2765 mg/L) were again in line with previous studies reporting a MIC of either 1920 mg/L [11] or 3250 mg/L [24].

In general, the relative bactericidal order of the active disinfectant components (benzalkoniumchloride > formaldehyde > glutaraldehyde) is also similar to that reported in both the latter studies [11, 24]. Small variations between results of susceptibility studies exist which can be attributed to the difference in bacteriological methods (broth dilution vs. agar dilution), media (TSB vs. MHB) and plate material (polypropylene vs. polystyrene) [25]. Therefore, standardisation of the MIC and MBC determination for disinfectants is needed to be able to survey these susceptibilities. In addition, it would be interesting to collate data from worldwide sources in a public database allowing to identify the distribution and set cut-off values.

When comparing the MICs and MBCs with in-use concentrations of the respective active components in veterinary disinfection products (e.g. in Virocid^®^ or CID20^®^), it was found that the MIC and MBC values for benzalkoniumchloride and formaldehyde were considerably lower assuming that the recommended concentrations of veterinary disinfection products are high enough to reduce the bacterial flora with 5 log colony forming units (CFU). In contrast, the MICs and MBCs for glutaraldehyde were much higher than the glutaraldehyde concentration used in veterinary disinfection products (e.g. in Virocid^®^ or CID20^®^). For the latter active disinfectant component, the use of a nutrient-rich medium like TSB in the MIC and MBC assays could be the reason for the high MICs and MBCs due to the reaction of glutaraldehyde with constituents of the growth medium [26]. Moreover, in the latter cross-sectional study glutaraldehyde was never used independently and is as far as we know always used in combination with QACs (e.g. benzalkoniumchloride) which has a synergistic biocidal effect (Maris, 1995). Finally, a commercially product (D50^®^), being a formulation of peracetic acid and hydrogen peroxide, was also tested in the current study. Our research group already demonstrated a MBC to D50 of 1% (10 mL/L) for *Enterobacteriaceae* isolates, although *E. coli* was not included in this previous study [12]. The MIC and MBC results for D50 in the current study were lower (between 1.25 to 5 mL/L) compared to the results of Luyckx et al. (2017). As this formulation of peracetic acid and hydrogen peroxide is a ready-to-use disinfectant for veterinary disinfection purposes, it can be concluded that the recommended concentration of 0.5% (5 mL/L) is just sufficient to kill our field isolates.

With the exception of the commercial disinfectant D50, single active components of disinfectants were used because the knowledge in case of reduced susceptibility to active components is the basis for understanding reduced susceptibility to commercial disinfection products, which are in most cases combinations of active components. However, none of the field isolates survived in-use concentrations of formaldehyde, benzalkoniumchloride and formulation of peracetic acid and hydrogen peroxide, which indicates that the proper use of disinfectants under practical conditions gives no indications for the selection for disinfectant resistance.

### Association between disinfectant use and antibiotic resistance prevalence

Previously, in vitro studies have shown an increase in antibiotic MICs after repeated sub-culturing of bacteria in subinhibitory concentrations of commercial disinfectants [7, 9] or active components [6]. Several other studies have found an association between decreased disinfectant susceptibility and antibiotic resistance. Still, these are results of non-standardized in vitro tests which do not provide information about the possible relation between disinfectant use and antibiotics resistance under practical conditions. Therefore, the effect of disinfectant use on antibiotic and disinfectant susceptibility of *E. coli* isolated from environmental samples after C&D was investigated in the current study. No significant positive associations were found between the use of active disinfectant components and antibiotic resistance. Remarkably, significant negative associations were found between the use of peracetic acid and hydrogen peroxide containing disinfectants and ampicillin, ciprofloxacin and tetracycline resistance in broiler production. These results suggest that the use of disinfectants containing this combination of active components would select for more susceptible *E. coli* bacteria. In literature, recent correlation studies performed with similar active components investigated associations between biocide susceptibility and antibiotic susceptibility**;** for peracetic acid and hydrogen peroxide containing disinfectants, no correlation between antibiotic resistance and MICs for peracetic acid and hydrogen peroxide containing products [27] or even a negative correlation between the susceptibility to hydrogen peroxide and antibiotic resistance to bramycin and aztreonam has been found [15]_,_ which is in line with our results. Nonetheless, a biological explanation for these observations is lacking. Furthermore, only 2 out of 25 broiler farms used a peracetic acid and hydrogen peroxide containing disinfectant. Therefore, future research on a larger number of farms and with a greater diversity in disinfection applications is warranted to further investigate these associations.

## Conclusions

As the *E. coli* field isolates showed a comparable antibiotic resistance profile with previous antibiotic resistance studies on fecal *E. coli* and because the disinfectant susceptibility results were homogenously distributed, it can be concluded that the *E. coli* strains found after C&D did not survive disinfection due to resistance but were still present due to inadequate C&D. Furthermore, all *E. coli* field isolates from broiler houses and pig nursery units were susceptible to in-use concentrations of formaldehyde, benzalkoniumchloride and formulation of peracetic acid and hydrogen peroxide, indicating that the proper use of disinfectants under practical conditions did not select for disinfectant resistance. Finally, the results of this study showed that there are no indications for the selection of antibiotic resistant bacteria through the use of disinfectants in agricultural environments.

## Methods

### Selection of farms

Belgian broiler and pig farms were randomly selected from the Belgian Identification and Registration (I&R) database by generating a list of random numbers via Excel which were linked to the farm list. The only selection criterion for broiler farms was that the flock contained at least 10,000 animals to be representative for the average practice situation. For pig farms the selection criteria were ‘farrow-to-finish’ or ‘feeder-to-finish’ types, and required the presence of piglets, sows and fattening pigs. A total of ca. 100 and 120 randomly selected broiler and pig farms respectively were invited by e-mail to participate. About a week later farmers were contacted by telephone and were asked whether they were willing to participate. Twenty-five broiler houses (flock size between 13,500 and 50,900 chicks) and 21 pig farms (pig nursery units consisted of 54 to 936 piglets) were visited once between March 2015 and July 2016. During these visits samples were taken and the farmer was interviewed face-to-face using a standardized questionnaire.

### Questionnaire design

The questionnaire consisted of open and closed questions and covered several aspects regarding flock and herd characteristics, biosecurity, cleaning and disinfection practices and antimicrobial consumption. Completion time for the questionnaire took about one and a half hour.

#### Collection of flock and herd data

For broilers, data were collected regarding flock size, flock slaughter age and flock slaughter weight, as well as the yearly average flock size, average number of flocks and average slaughter weight. Questions for the sampled pig nursery units concerned the number of weaner pigs, age and weight when entering the nursery units, and age and weight at relocation to the fattening unit. The questionnaires developed for this study are provided in additional files (see additional files 2 and 3).

#### Quantification of biosecurity status

Evaluation of the biosecurity status in the broiler farms and pig herds was obtained using a previously defined questionnaire Biocheck.Ugent® available as an online tool: http://www.biocheck.ugent.be/biocheck.php (Biocheck.Ugent poultry: version 2.1; Biocheck.Ugent pigs: version 2.0). After putting the data into the Biocheck.Ugent tool, the external and internal biosecurity scores and their appropriate sub-categories were calculated and summarized into a report. The overall score was calculated as the mean of the external and internal biosecurity score.

#### Cleaning and disinfection practices

Questions regarding the applied cleaning and disinfection protocol were also asked and listed in additional files (see Additional files 2 and 3). For every sampled poultry or pig farm, the used disinfectants were recorded and the presence or absence of active components were listed into a Microsoft Excel spreadsheet (Microsoft, 2016) via a binary system. These active components of disinfectants were quaternary ammonium compounds (QACs), glutaraldehyde (GA), formaldehyde (F), peracetic acid (PA), hydrogen peroxide (H_2_O_2_) and other components (e.g. chlorine and potassium peroxymonosulfate). Quaternary ammonium compounds and glutaraldehyde (QACs-GA) and hydrogen peroxide and peracetic acid (PA-H_2_O_2_) were listed together as these active components are generally combined.

#### Quantification of antibiotic use

Data on antibiotic use for group treatments at the sampled animal houses were also obtained via prescriptions and order forms. For each group treatment, the product name, the amount of administration and the age (days) and weight (kg) of the treated animals were recorded. Quantification of drug use was done by determining the treatment incidence (TI_100_) defined as the number of treatment days per 100 days or the % of treatment days [28]. The following formula was used to calculate the TI_100_ per production round:

$$ TI100\ \left[ round\right]=\frac{total\ amount\ of\ antimicrobial\ administered\ (mg)}{DDD\ \left(\frac{mg}{kg}\right)\times animal\ amount\ (kg)\times number\ of\ days\  at\  risk}\times LA\  factor\times 100 $$

In this equation, the Defined Daily Dose (DDD) is the nationally determined average maintenance dose per day and per kg animal of a specific antibiotic, the total animal amount is calculated as the number of animals multiplied by the average weight of the animals at the moment of treatment and the ‘number of days at risk’ is the duration of the production period considered. The Long Acting factor (LA factor) is used for long acting products and takes a longer duration of action into account [29].

### Collection of samples

Sampling was performed 24 h ± 4 h after cleaning and disinfection with sponge sticks (3 M, SSL100, St-Paul, USA) moistened with 10 mL Dey Engley Neutralizing Broth (DE broth, Sigma Aldrich, D3435, St-Louis, USA). For each broiler house six different types of sampling points were each sampled eight times: floor, floor crack, drain hole, air inlet, drinking cups and pipes (based on previous research from our group (Luyckx et al., (2015) [30] showing the highest percentage of swab samples positive for *E. coli* after cleaning and disinfection at 12 sampling locations), resulting in 48 swab samples per broiler house. A surface of 625 cm^2^ was swabbed whenever possible. Since the surface of the drinking cups was smaller than 625 cm^2^, five drinking cups were sampled with the same sponge stick. For pig nursery units, in total four pens were sampled. At each pen, six different sampling locations were swabbed: floor, concrete wall, synthetic wall, feeding trough, drinking nipples and pipes, resulting in 24 environmental swab samples per pig nursery unit. A surface of 625 cm^2^ was swabbed whenever possible. Since the pens of a pig nursery unit contains a drinking unit ranging from 1 to 10 nipples, a maximum of 2 nipples per pen was swabbed whenever possible and analysed as one sample. Sampling of pig nursery units was also based on previous work from our group by Luyckx et al. (2016) [31].

### Detection and isolation of *Escherichia coli*

After sampling, swabs were transported to the lab in a cool box with ice packs. Upon their arrival in the lab (± 2 h after sampling), 10 mL of Buffered Peptone Water (BPW, Oxoid, CM0509, Basingstoke, Hampshire, England) was immediately added to each sample, homogenized by a Masticator (IUL instruments, S.A., Barcelona, Spain) and incubated for 24 h at 37 °C for enrichment of *E. coli*. After incubation, 10 μL of the enriched BPW fraction was plated on Rapid’*E. coli* 2 agar plates (Biorad, 356–4024, Marnes-la-Coquettes, France) and incubated at 44 °C for 24 h. From positive Rapid’*E. coli* 2 plates purified isolates were obtained and stored at − 80 °C on brain heart infusion (BHI, Oxoid, CM1032) supplemented with 15% (v/v) glycerol.

### Antibiotic susceptibility testing

For each positive sample from detection plates, one *E. coli* isolate was collected for antibiotic susceptibility testing. Antibiotic susceptibility testing was performed using a microdilution method (Sensititre). Fourteen different antimicrobial agents specified by the EFSA were tested using a custom plate format (Sensititre® plate: EUVSEC): sulfamethoxazole (8–1024 μg/mL), trimethoprim (0.25–32 μg/mL), ciprofloxacin (0.015–8 μg/mL), tetracycline (2–64 μg/mL), meropenem (0.03–16 μg/mL), azithromycin (2–64 μg/mL), nalidixic acid (4–128 μg/mL), cefotaxime (0.25–4 μg/mL), chloramphenicol (8–128 μg/mL), tigecycline (0.25–8 μg/mL), ceftazidime (0.5–8 μg/mL), colistin (1–16 μg/mL), ampicillin (1–64 μg/mL) andgentamicin 0.5–32 μg/mL). Inoculum was prepared by picking ca. three to five colonies from an overnight Plate Count Agar (PCA, Oxoid, CM0325) plate and diluting/suspending in 5 mL demineralized water (Sterile destilled water, Thermo Scientific, YT3339) to a Mc Farland of 0.5 (~ 10^8^ CFU/mL) using a Nephelometer® (Thermo Scientific, V3011) to standardize inoculum density/turbidity. Cell suspension was further diluted by dispersing 10 μL in 11 mL cation-adjusted Mueller-Hinton broth (MHB) with TES buffer (CAMHB, Thermo Scientific, YT3462). 50 μL of the inoculated MHB was transferred to each well of the EUVSEC-plate (~ 5 × 10^4^ CFU/mL). EUVSEC-plates were incubated at 37 °C for 24 h and MIC was defined as the lowest concentration without visible growth using the Sensititre™ Vizion™ System (Thermo Scientific, V2020) and the Sensititre™ Windows® Software System (SWIN™). A reference strain (*E. coli* ATCC 25922) was taken along with each batch of susceptibility tests as internal quality control.

To check the inoculum concentration and purity, 10 μL from the positive control well was transferred in 10 mL demineralized water and thoroughly mixed prior to transferring 100 μL of the inoculum to a PCA-plate, spread with a Drigalski spatula and incubation at 37 °C.

#### *Escherichia coli* antibiotic resistance profile

For each isolate and each antimicrobial substance, the MIC was read and converted in binary qualitative values (wild type, further referred to as susceptible (S) and non-wild type further referred to as resistant (R)) based on the epidemiological cut-off values (ECOFF) (R: MIC > ECOFF, S: MIC ≤ ECOFF) defined by EUCAST (https://mic.eucast.org/Eucast2/). For azithromycin no ECOFF was available in the EUCAST-database so the cut-off 16 mg/L used by EFSA [32] was applied.

### Disinfectant susceptibility testing

#### Isolate and disinfectant selection

For each sampled poultry house and pig nursery unit three (if available) *E. coli* isolates from distinct sampling locations and with the highest number of antimicrobial resistances were selected in order to study the possible decreased disinfectant susceptibility in the more antibiotic resistant population. A total of 57 poultry and 61 pig isolates were examined. Based on the results of the questionnaire and on research from our group by Maertens et al. (2018) [18], active components most frequently occurring in disinfectants used in the sampled poultry houses and pig nursery units were selected, being: alkyldimethylbenzylammoniumchloride (BKC, > 95%, Sigma Aldrich) which is a QAC, formaldehyde (F, 35% vol/vol in H_2_O, Sigma Aldrich), glutaraldehyde (GA, 50% w/v in H_2_O, Sigma Aldrich) and a chemically stable formulation of peracetic acid (PA, 55 g/L) and hydrogen peroxide (H_2_O_2_, 220 g/L) (D50®, CID LINES, Ieper, Belgium) as H_2_O_2_ rapidly degrades into water and oxygen and PA can decompose to acetic acid and oxygen [1].

#### Inoculum preparation

The selected isolates were cultured on PCA at 37 °C for 24 h. Per agar plate, one colony was picked and used to inoculate 10 mL of Tryptone Soya Broth (TSB, Oxoid, CM0129) and grown at 37 °C for 16 h to obtain fresh liquid cultures. Subsequently, liquid cultures were centrifuged at 5000 *g* for 10 min and the supernatant was discarded. The remaining pellet was resuspended in 10 mL Ringers solution (Oxoid, BR0052). Next, inocula were diluted with Ringer solution to an optical density at 600 nm (OD600) corresponding with a viable count of 1–5 × 10^8^ CFU/mL. To control the inoculum concentration, enumerations on PCA were carried out by using a spiral plater (Eddy Jet, IUL instruments, S.A., Barcelona, Spain).

#### Reproducibility of the data

To check the reproducibility and repeatability of the assay, eight isolates were tested in triplicate, on two different occasions. From then on, each isolate was tested only once.

#### Minimum inhibitory concentration (MIC)

The MICs of each active component (BKC, F and GA) or given formulation (D50) for the selected isolates were determined with a broth microdilution method based on the method described by Knapp et al. (2015) [33].

A 96-well microtiter plate with U-shaped wells (Novolab, A19652) was filled with 50 μL TSB containing twofold dilutions of the active component or formulation. Fifty microliters of the field isolates (1–5 × 10^8^ CFU bacterial /mL) were added to the TSB in the microtiter plate, resulting in a total volume of 100 μL. Final concentration ranges were as follows: 0.213–0.007 g/L BKC, 1.480–0.046 mL/L F, 20–0.625 mL/L GA and 20–0.125 mL/L D50. As a positive control, 50 μL of each bacterial suspension was added to 50 μL TSB without disinfectant. To check for possible contamination, wells without bacterial suspension and disinfectant served as blank. After inoculation, plates were incubated for 24 h in a shaking incubator (100 rpm) at 37 °C. After incubation, the MICs were read. The MIC was defined as the lowest concentration of active components or formulation where no growth was visually observed. In every experiment the *E. coli* reference strains for antibiotic susceptibility (ATCC 25922) and disinfectant susceptibility (ATCC 10536) were used as controls.

#### Minimum bactericidal concentration (MBC)

After determining the MIC, 20 μL of the cell suspension in the microtiter plate was transferred to a new 96-well round-bottom microtiter plate filled with 180 μL DE broth for 5 min. Subsequently, 12.5 μL of each well was spotted on PCA-plates. Plates were incubated at 37 °C for 24 h and the MBC was determined. The MBC was defined as the lowest concentration where no visible growth on the agar plate was observed (~ 5 log CFU reduction).

##### Data analysis

For both animal categories the antibiotic resistance prevalence and the accompanying 95% confidence interval was calculated for each antibiotic based on the standard error of the binomial distribution in Microsoft Excel (Microsoft, 2016). The association between active components used (absent = 0, present = 1) during disinfection and antibiotic resistance at each farm was tested by means of binary logistic regression analysis taking the corresponding antibiotic use (TI100) into account as co-variable. First, the independent variables (‘use of QACs-GA’, ‘use of F’, ‘use of PA-H_2_O_2_’ and ‘use of other active components’) were tested univariable for all antibiotics (n = 13 by combining sulfamethoxazole and trimethoprim resistance). Those variables with univariable P-values of < 0.20 were retained for further analysis in a multivariable model. Subsequently, with the retained variables, a multivariable logistic regression model was constructed using the stepwise backward elimination procedure starting with the global model and gradually excluding all non-significant factors. Multivariate binary logistic regression models were used for each antibiotic. As multiple models were tested to evaluate the effect of the different active components on the different types of antibiotic resistance a bonferroni correction for multiple testing was performed. P-values ≤0.0038 (after Bonferroni correction) were considered as significant. All statistics were performed using SPSS Statistics 25.0 (IBM Corporation, Armonk, NY).

## Additional files

Additional file 1: **Table S1.** Example set of antibiotic resistance profiles of *Escherichia coli* derived from broiler and pig farms using different active disinfectant components. It describes the difference in antibiotic resistance profiles between *E. coli* strains isolated from different locations at the same farm. Furthermore, the active components used during disinfection at the respective farms are listed. (DOCX 23 kb)

Additional file 2: Questionnaire 1: Measuring both cleaning and disinfection practices and antibiotic usage at broiler farms. It describes the set of questions asked to all participating poultry farmers related to the applied cleaning and disinfection protocol and antibiotic use. (DOCX 68 kb)

Additional file 3: Questionnaire 2: Measuring both cleaning and disinfection practices and antibiotic usage at pig farms. It describes the set of questions asked to all participating pig farmers related to the applied cleaning and disinfection protocol and antibiotic use. (DOCX 68 kb)

## Data Availability

The datasets used and analysed during the current study are available from the corresponding author on reasonable request.

## Abbreviations

BKC: Benzalkoniumchloride
BPW: Buffered Peptone Water
C&D: Cleaning and disinfection
CFU: Colony forming units
DE broth: Dey Engley Neutralizing Broth
*E. coli*: *Escherichia coli*
ECOFF: Epidemiological cut-off
F: Formaldehyde
GA: Glutaraldehyde
H_2_O_2_: Hydrogen peroxide
ILVO: Flanders Research Institute for Agriculture, Fisheries and Food
MBC: Minimum Bactericidal Concentration
MHB: Mueller-Hinton broth
MIC: Minimum Inhibitory Concentration
PA: Peracetic acid
PCA: Plate Count Agar
QAC: Quaternary ammonium compound
TSB: Trypton Soya Broth
