# Supplementary material for: Limited association between disinfectant use and either antibiotic or disinfectant susceptibility of Escherichia coli in both poultry and pig husbandry
Source: BMC Vet Res. 2019 Sep 2;15:310. doi: 10.1186/s12917-019-2044-0 (PMC6721165; doi:10.1186/s12917-019-2044-0)
Supplement: Supplementary file 1 — Table S1. Example set of antibiotic resistance profiles of Escherichia coli derived from broiler and pig farms using different active disinfectant components. It describes the difference in antibiotic resistance profiles between E. coli strains isolated from different locations at the same farm. Furthermore, the active components used during disinfection at the respective farms are listed. (DOCX 23 kb) [file 12917_2019_2044_MOESM1_ESM.docx]

Supplementary Table 1: Example set of antibiotic resistance profiles of Escherichia coli derived from broiler and pig farms using different active disinfectant components.

| **Origin** | **Farm** | **Active disinfectant components** | **Sampling location** | ***E. coli* isolate** | **Ampicillin** | **Azithromycin** | **Cefotaxime** | **Ceftazidime** | **Chloramphenicol** | **Ciprofloxacin** | **Colistin** | **Gentamicin** | **Meropenem** | **Nalidixic Acid** | **Sulfamethoxazole** | **Tetracycline** | **Tigecycline** | **Trimethoprim** | **Number of antibiotic resistances** |
| --- | --- | --- | --- | --- | --- | --- | --- | --- | --- | --- | --- | --- | --- | --- | --- | --- | --- | --- | --- |
| **Broiler houses** | A | Q-F-GL | Drain hole | A66 | 1 | 0 | 0 | 0 | 1 | 1 | 0 | 0 | 0 | 1 | 1 | 1 | 0 | 1 | 7 |
|  |  |  | Drinking cups | A46 | 1 | 0 | 1 | 1 | 1 | 1 | 0 | 0 | 0 | 1 | 1 | 1 | 0 | 0 | 8 |
|  |  |  | Pipes | A16 | 1 | 0 | 0 | 0 | 0 | 1 | 0 | 0 | 0 | 1 | 0 | 1 | 0 | 0 | 4 |
|  | C | Q-GL | Drinking cups | C45 | 0 | 0 | 0 | 0 | 0 | 1 | 0 | 0 | 0 | 1 | 0 | 0 | 0 | 0 | 2 |
|  |  |  | Floor | C25 | 0 | 0 | 0 | 1 | 0 | 0 | 0 | 0 | 0 | 0 | 1 | 1 | 0 | 0 | 3 |
|  |  |  | Floor crack | C1 | 1 | 0 | 0 | 0 | 0 | 0 | 0 | 0 | 0 | 0 | 1 | 1 | 0 | 1 | 4 |
|  | D | Other | Air inlet | D2 | 1 | 0 | 0 | 0 | 0 | 0 | 0 | 0 | 0 | 0 | 1 | 0 | 0 | 1 | 3 |
|  |  |  | Drain hole | D6 | 1 | 0 | 1 | 1 | 1 | 1 | 0 | 0 | 0 | 1 | 1 | 1 | 0 | 0 | 8 |
|  |  |  | Floor crack | D8 | 1 | 0 | 0 | 0 | 0 | 0 | 0 | 0 | 0 | 0 | 0 | 0 | 0 | 0 | 1 |
|  | K | Q-GL | Drain hole | K1 | 0 | 0 | 0 | 0 | 0 | 1 | 0 | 0 | 0 | 1 | 1 | 1 | 0 | 1 | 5 |
|  |  |  | Drinking cups | K8 | 1 | 0 | 1 | 1 | 0 | 1 | 0 | 0 | 0 | 1 | 1 | 1 | 1 | 1 | 9 |
|  |  |  | Drain hole | K11 | 1 | 0 | 0 | 0 | 0 | 0 | 0 | 0 | 0 | 0 | 1 | 0 | 0 | 1 | 3 |
|  | O | F | Floor crack | O1 | 1 | 0 | 0 | 0 | 0 | 1 | 0 | 0 | 0 | 1 | 1 | 0 | 0 | 1 | 5 |
|  | P | F | Drain hole | P1 | 1 | 0 | 0 | 0 | 1 | 1 | 0 | 0 | 0 | 0 | 1 | 1 | 0 | 1 | 6 |
|  |  |  | Floor crack | P16 | 1 | 0 | 0 | 0 | 1 | 1 | 0 | 0 | 0 | 1 | 1 | 1 | 0 | 1 | 7 |
|  |  |  | Floor crack | P26 | 1 | 0 | 0 | 0 | 0 | 1 | 0 | 0 | 0 | 1 | 1 | 1 | 0 | 1 | 6 |
|  | Q | P+P | Drain hole | Q21 | 1 | 0 | 0 | 0 | 0 | 1 | 0 | 0 | 0 | 1 | 0 | 1 | 0 | 1 | 5 |
|  |  |  | Air inlet | Q51 | 1 | 0 | 0 | 0 | 0 | 0 | 0 | 0 | 0 | 0 | 1 | 1 | 0 | 1 | 4 |
|  |  |  | Floor crack | Q79 | 1 | 0 | 1 | 1 | 1 | 0 | 0 | 0 | 0 | 0 | 1 | 1 | 0 | 0 | 6 |
|  | U | P+P | Drain hole | U13 | 1 | 0 | 0 | 0 | 0 | 1 | 0 | 0 | 0 | 1 | 1 | 1 | 0 | 1 | 6 |
|  |  |  | Drinking nipples | U34 | 1 | 0 | 0 | 0 | 0 | 0 | 0 | 0 | 0 | 0 | 1 | 0 | 0 | 1 | 3 |
|  |  |  | Floor crack | U37 | 1 | 0 | 0 | 0 | 0 | 1 | 0 | 0 | 0 | 0 | 1 | 1 | 0 | 1 | 5 |
| **Pig nursery units** | A | Other | Pipes | A100 | 1 | 0 | 0 | 0 | 1 | 0 | 0 | 1 | 0 | 0 | 1 | 1 | 0 | 1 | 6 |
|  |  |  | Concrete wall | A86 | 1 | 0 | 0 | 0 | 1 | 0 | 0 | 0 | 0 | 0 | 1 | 1 | 0 | 1 | 5 |
|  |  |  | Synthetic wall | A39 | 1 | 0 | 0 | 0 | 0 | 1 | 0 | 0 | 0 | 1 | 1 | 1 | 0 | 1 | 6 |
|  | D | P+P | Drinking nipples | D21 | 1 | 0 | 0 | 0 | 1 | 0 | 0 | 0 | 0 | 0 | 1 | 0 | 0 | 1 | 4 |
|  |  |  | Synthetic wall | D41 | 0 | 0 | 0 | 0 | 1 | 0 | 0 | 0 | 0 | 0 | 1 | 1 | 0 | 1 | 4 |
|  |  |  | Feeding trough | D46 | 1 | 0 | 0 | 0 | 1 | 0 | 0 | 0 | 0 | 0 | 1 | 1 | 0 | 1 | 5 |
|  | E | P+P | Floor | E1 | 1 | 0 | 0 | 0 | 0 | 1 | 0 | 0 | 0 | 1 | 1 | 1 | 0 | 1 | 6 |
|  |  |  | Drinking nipples | E16 | 1 | 0 | 0 | 0 | 1 | 1 | 0 | 1 | 0 | 1 | 1 | 1 | 0 | 1 | 8 |
|  |  |  | Feeding trough | E31 | 1 | 0 | 0 | 0 | 1 | 1 | 0 | 1 | 0 | 1 | 1 | 1 | 0 | 1 | 8 |
|  | P | Q-GL | Drinking nipples | P19 | 1 | 0 | 0 | 0 | 1 | 0 | 0 | 0 | 0 | 0 | 1 | 1 | 0 | 1 | 5 |
|  |  |  | Pipes | P13 | 0 | 0 | 0 | 0 | 0 | 1 | 0 | 0 | 0 | 1 | 1 | 0 | 0 | 1 | 4 |
|  |  |  | Feeding trough | P9 | 1 | 0 | 0 | 0 | 1 | 0 | 0 | 1 | 0 | 0 | 1 | 1 | 0 | 1 | 6 |
|  | S | Q-F-GL | Pipes | S3 | 1 | 0 | 0 | 0 | 0 | 1 | 0 | 0 | 0 | 1 | 1 | 1 | 0 | 1 | 6 |
|  |  |  | Floor | S1 | 1 | 0 | 0 | 0 | 1 | 0 | 0 | 0 | 0 | 0 | 1 | 1 | 0 | 1 | 5 |
|  |  |  | Feeding trough | S11 | 1 | 0 | 0 | 0 | 0 | 0 | 0 | 1 | 0 | 0 | 1 | 1 | 0 | 1 | 5 |

F, formaldehyde; PA, peracetic acid; H_2_O_2_, hydrogen peroxide; QAC, quaternary ammonium compound; GA, glutaraldehyde
