# Supplementary material for: Limited association between disinfectant use and either antibiotic or disinfectant susceptibility of Escherichia coli in both poultry and pig husbandry
Source: BMC Vet Res. 2019 Sep 2;15:310. doi: 10.1186/s12917-019-2044-0 (PMC6721165; doi:10.1186/s12917-019-2044-0)
Supplement: Supplementary file 2 — Questionnaire 1: Measuring both cleaning and disinfection practices and antibiotic usage at broiler farms. It describes the set of questions asked to all participating poultry farmers related to the applied cleaning and disinfection protocol and antibiotic use. (DOCX 68 kb) [file 12917_2019_2044_MOESM2_ESM.docx]

Questionnaire 1: Measuring both cleaning and disinfection practices and antibiotic usage at broiler farms

# General information

| Date of survey completion (dd/mm/yyyy) | / / |
| --- | --- |

## Administrative information of the farmer

| First and last name |  |
| --- | --- |
| Phone number |  |
| Email adress |  |

## Administrative information of the farm

| Farm adress | Street: |
| --- | --- |
|  | Postal code: City: |

# Collection of average flock data

1. Yearly average flock size?..…..……………………………………………………………………
2. Yearly average number of flocks? ………………………………………………………………..
3. Average slaughter weight? …………………………………………………………………………

# Flock data of the sampled broiler house

1. Start date of the (last) production round: / /
2. End date of the (last) production round: / /
3. Number of broiler chicks entering the broiler house?.....…….…….………………………………
4. Age of broiler chicks entering the broiler house (days)?.....…………….…………… ……………
5. Weight of broiler chicks entering the broiler house?.....……….…..…………………………….…
6. Slaughter age of the broiler chicks?…………… …..………….……….……………………………
7. Number of broiler chicks at slaughter age?..…………………………………………………...……
8. Weight of broiler chicks at slaughter age?....…………... ………………………………………...…

# Cleaning and disinfection of the sampled broiler house

## Vacancy period

1. How long is the vacancy period? ……………………………………………..………………..…..
2. On which day during the vacancy period does dry cleaning take place? ………………………….
3. On which day during the vacancy period does wet cleaning take place? …………………………
4. On which day during the vacancy period does disinfection take place? ………………………..…
5. How long do the stables remain vacant after disinfection? ……………………………………..….

## Cleaning

**Dry cleaning**

1. Does each broiler house get dry cleaned after each production cycle?

- After every production cycle
- Sometimes; how often? ................................
- Never

1. Who does the dry cleaning?

- Farmer
- External company

1. Describe the dry cleaning steps in detail.

|  | Step | Tools / Method | Location(s) |
| --- | --- | --- | --- |
| 1 |  |  |  |
| 2 |  |  |  |
| 3 |  |  |  |
| 4 |  |  |  |

**Wet cleaning**

1. Does each broiler house get wet cleaned after each production cycle?

- After every production cycle
- Sometimes; how often? ...................
- Never

1. Who does the wet cleaning?

- Farmer
- External company

1. Describe the soaking and/or cleaning steps.

|  | Step | Tools / Method | Cleaning product used (product name, concentration, …) | Location(s) |
| --- | --- | --- | --- | --- |
| 1 |  |  |  |  |
| 2 |  |  |  |  |
| 3 |  |  |  |  |

**Rinsing**

1. Is the cleaning product rinsed off? ………………………………………………….……………..

## DISINFECTION

1. Is the broiler house disinfected after each production cycle?

- After every production cycle
- Sometimes; how often does disinfection takes place? .........................................................
- Never

1. Who performs the disinfection?

- Farmer
- External company

1. Describe the disinfection steps.

|  | Step | Method ^[[1]](#footnote-1)^ / tools | Disinfection product used (product name, concentration, …) | Location(s) |
| --- | --- | --- | --- | --- |
| 1 |  |  |  |  |
| 2 |  |  |  |  |
| 3 |  |  |  |  |
| 4 |  |  |  |  |

1. Is there a difference in the applied disinfection between the two last disinfections and the current disinfection? ………………………………………………………………………….……………..

**Rinsing**

1. Is the disinfection product rinsed off? ……….……………………………………….……………..

# Antibiotic use: group treatments at the sampled animal house

| **Treatment** | **Number of treated animals** | **Product name and concentration** | **Total administered amount** | **Administration route (feed, water, ..)** | **Weight at treatment**  **(kg)** | **Age at treatment**  **(days)** |
| --- | --- | --- | --- | --- | --- | --- |
| 1 |  |  |  |  |  |  |
| 2 |  |  |  |  |  |  |
| 3 |  |  |  |  |  |  |
| 4 |  |  |  |  |  |  |

1. fogging, spraying or foaming [↑](#footnote-ref-1)
