# Supplementary material for: Limited association between disinfectant use and either antibiotic or disinfectant susceptibility of Escherichia coli in both poultry and pig husbandry
Source: BMC Vet Res. 2019 Sep 2;15:310. doi: 10.1186/s12917-019-2044-0 (PMC6721165; doi:10.1186/s12917-019-2044-0)
Supplement: Supplementary file 3 — Questionnaire 2: Measuring both cleaning and disinfection practices and antibiotic usage at pig farms. It describes the set of questions asked to all participating pig farmers related to the applied cleaning and disinfection protocol and antibiotic use. (DOCX 68 kb) [file 12917_2019_2044_MOESM3_ESM.docx]

Questionnaire 2: Measuring both cleaning and disinfection practices and antibiotic usage at pig farms

# General information

| Date of survey completion (dd/mm/yyyy) | / / |
| --- | --- |

## Administrative information of the farmer

| First and last name |  |
| --- | --- |
| Phone number |  |
| Email adress |  |

## Administrative information of the farm

| Farm adress | Street: |
| --- | --- |
|  | Postal code: City: |

# Characteristics of the sampled pig nursery unit

1. How many pig nursery units does the sampled stable have? ................................................…….…
2. How many pens does the pig nursery unit have?......................................……....………….…..……
3. What is the surface area of the sampled pig nursery unit? ……………………………………….....

# Herd data of the sampled pig nursery unit

1. Start date of the (last) production round: / /
2. End date of the (last) production round: / /
3. Number of weaner pigs entering the nursery unit?.....…………..……………..……………………
4. Age of weaner pigs entering the nursery unit (days)?.....………..………..……..……………….…
5. Weight of weaner pigs entering the nursery unit?.....……………..…….…….……………….……
6. Number of weaner pigs when relocated to the fattening unit?..…………..…………………...……
7. Age of weaner pigs when relocated to the fattening unit (days)?.....…………………………….…
8. Weight of weaner pigs when relocated to the fattening unit?.....………………..……………….…

# Cleaning and disinfection of the sampled pig nursery unit

## Vacancy period

1. How long is the vacancy period? ……………………………………….……..………………..…..
2. On which day during the vacancy period does dry cleaning take place? ……………..…..……….
3. On which day during the vacancy period does wet cleaning take place? ……………….…………
4. On which day during the vacancy period does disinfection take place? ……...……………………
5. How long do the stables remain vacant after disinfection? ………………………………………….

## Cleaning

**Dry cleaning**

1. Does each pig nursery unit get dry cleaned after each production cycle?

- After every production cycle
- Sometimes; how often? ................................
- Never

1. Who does the dry cleaning?

- Farmer
- External company

1. Describe the dry cleaning steps in detail.

|  | Step | Tools / Method | Location(s) |
| --- | --- | --- | --- |
| 1 |  |  |  |
| 2 |  |  |  |
| 3 |  |  |  |
| 4 |  |  |  |

**Wet cleaning**

1. Does each pig nursery get wet cleaned after each production cycle?

- After every production cycle
- Sometimes; how often? ...................
- Never

1. Who does the wet cleaning?

- Farmer
- External company

1. Describe the soaking and/or cleaning steps.

|  | Step | Tools / Method | Cleaning product used (product name, concentration, …) | Location(s) |
| --- | --- | --- | --- | --- |
| 1 |  |  |  |  |
| 2 |  |  |  |  |
| 3 |  |  |  |  |
| 4 |  |  |  |  |

**Rinsing**

1. Is the cleaning product rinsed off? ……………………………………………….……………..

## DISINFECTION

1. Is the pig nursery unit disinfected after each production cycle?

- After every production cycle
- Sometimes; how often does disinfection takes place? .........................................................
- Never

1. Who performs the disinfection?

- Farmer
- External company

1. Describe the disinfection steps.

|  | Step | Method ^[[1]](#footnote-1)^ / tools | Disinfection product used (product name, concentration, …) | Location(s) |
| --- | --- | --- | --- | --- |
| 1 |  |  |  |  |
| 2 |  |  |  |  |
| 3 |  |  |  |  |
| 4 |  |  |  |  |

1. Is there a difference in the applied disinfection between the two last disinfections and the current disinfection? ………………………………………………………………………….……………..

**RINSING**

1. Is the disinfection product rinsed off? ………………………………………….……………..

# Antibiotic use: group treatments at the sampled animal houses

| **Treatment** | **Number of treated animals** | **Product name and concentration** | **Total administered amount** | **Administration route (feed, water, ..)** | **Weight at treatment**  **(kg)** | **Age at treatment**  **(days)** |
| --- | --- | --- | --- | --- | --- | --- |
| 1 |  |  |  |  |  |  |
| 2 |  |  |  |  |  |  |
| 3 |  |  |  |  |  |  |
| 4 |  |  |  |  |  |  |

1. fogging, spraying or foaming [↑](#footnote-ref-1)
